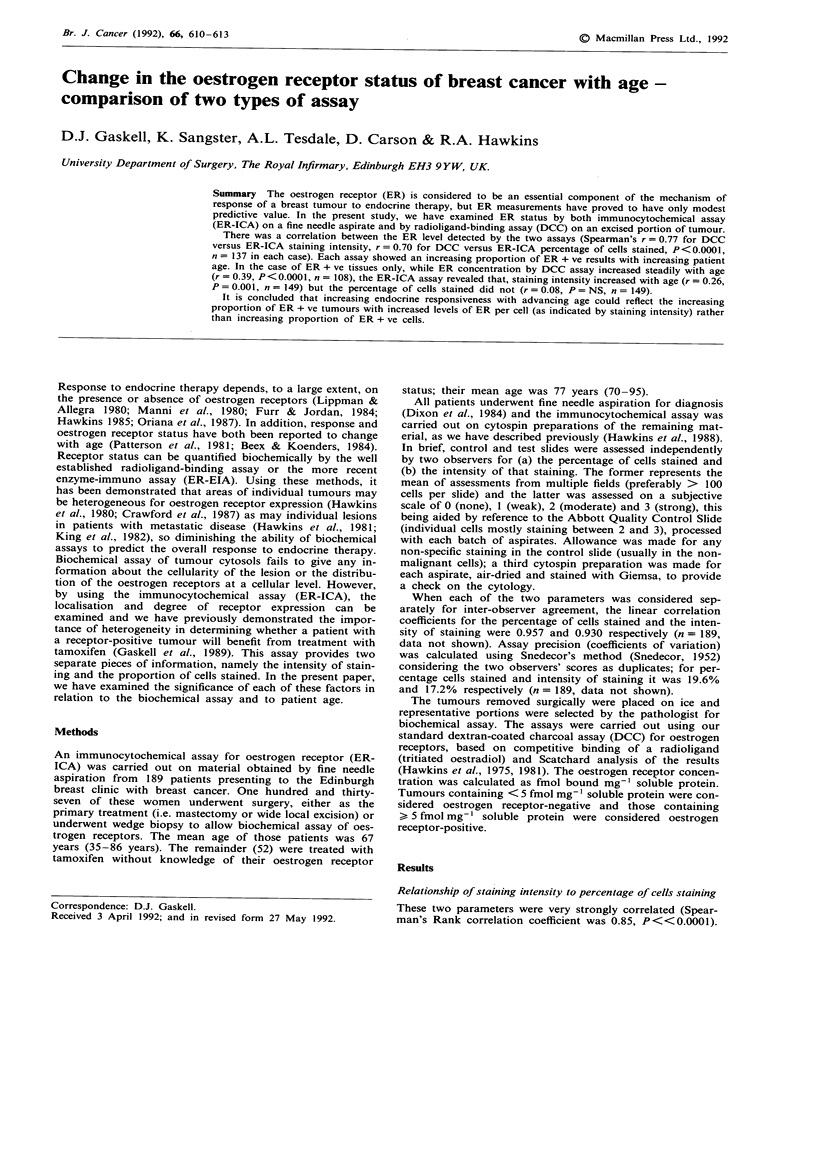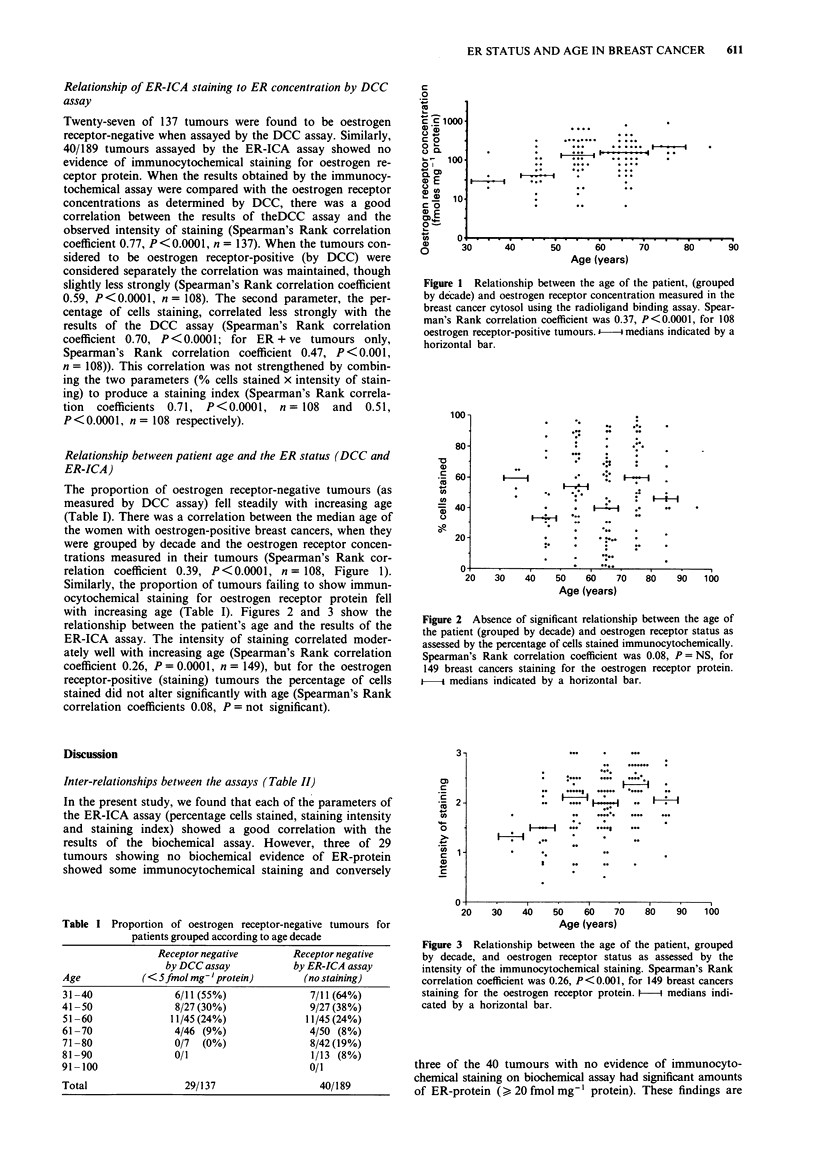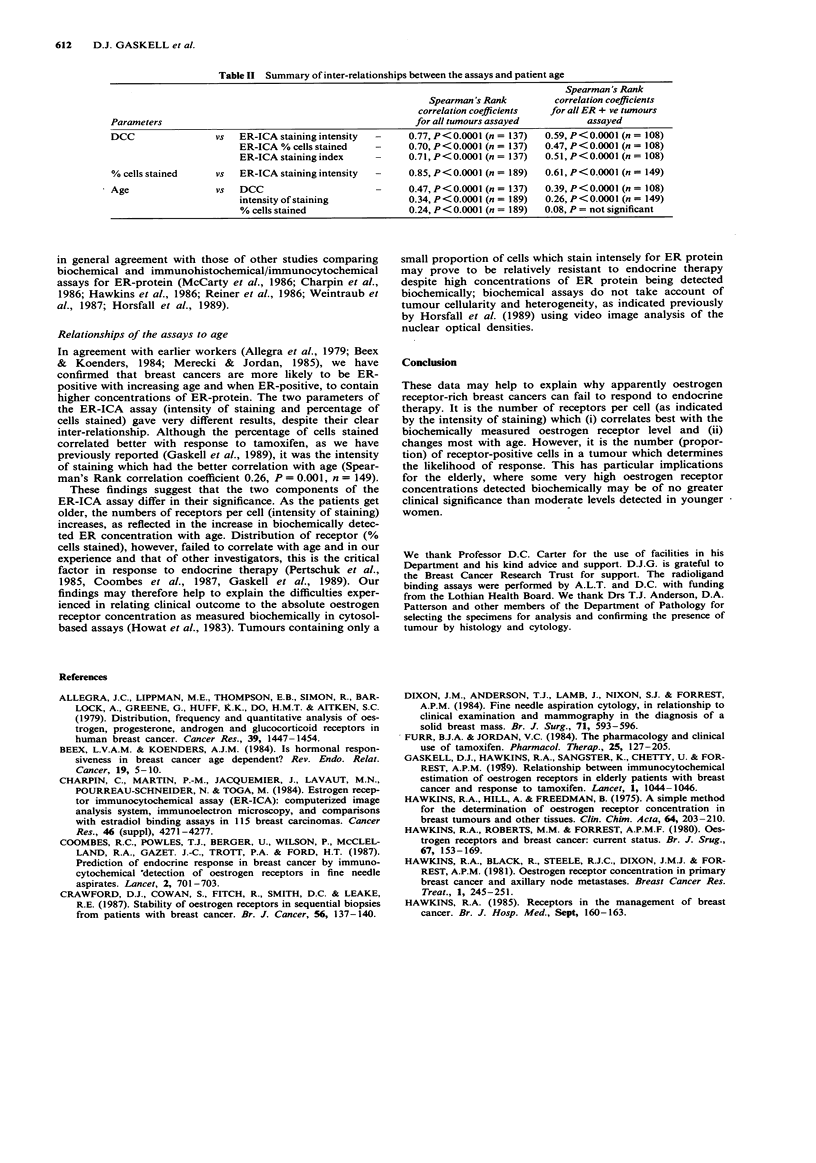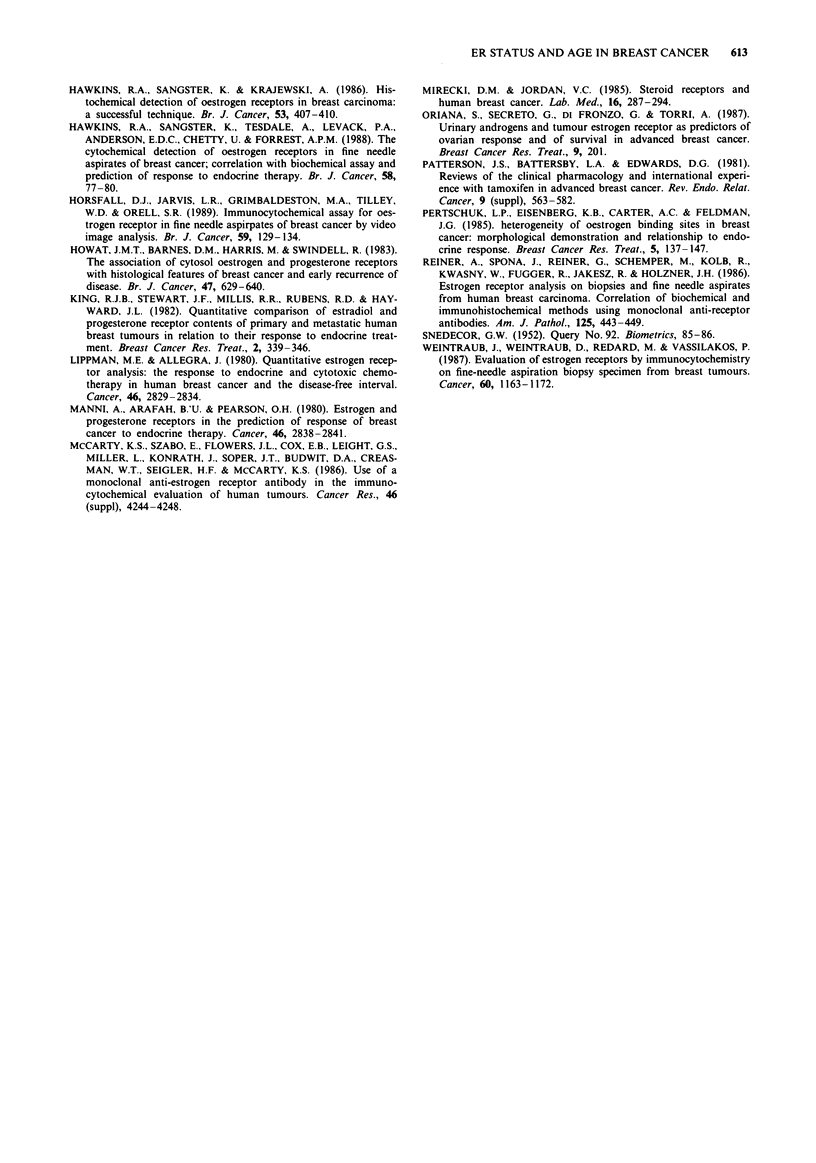# Change in the oestrogen receptor status of breast cancer with age--comparison of two types of assay.

**DOI:** 10.1038/bjc.1992.324

**Published:** 1992-10

**Authors:** D. J. Gaskell, K. Sangster, A. L. Tesdale, D. Carson, R. A. Hawkins

**Affiliations:** University Department of Surgery, Royal Infirmary, Edinburgh, UK.

## Abstract

The oestrogen receptor (ER) is considered to be an essential component of the mechanism of response of a breast tumour to endocrine therapy, but ER measurements have proved to have only modest predictive value. In the present study, we have examined ER status by both immunocytochemical assay (ER-ICA) on a fine needle aspirate and by radioligand-binding assay (DCC) on an excised portion of tumour. There was a correlation between the ER level detected by the two assays (Spearman's r = 0.77 for DCC versus ER-ICA staining intensity, r = 0.70 for DCC versus ER-ICA percentage of cells stained, P < 0.0001, n = 137 in each case). Each assay showed an increasing proportion of ER+ve results with increasing patient age. In the case of ER+ve tissues only, while ER concentration by DCC assay increased steadily with age (r = 0.39, P < 0.0001, n = 108), the ER-ICA assay revealed that, staining intensity increased with age (r = 0.26, P = 0.001, n = 149) but the percentage of cells stained did not (r = 0.08, P = NS, n = 149). It is concluded that increasing endocrine responsiveness with advancing age could reflect the increasing proportion of ER+ve tumours with increased levels of ER per cell (as indicated by staining intensity) rather than increasing proportion of ER+ve cells.


Br. J. Cancer (1992), 66, 610-613 © Macmillan Press Ltd., 1992

### Change in the oestrogen receptor status of breast cancer with age – comparison of two types of assay

D.J. Gaskell, K. Sangster, A.L. Tesdale, D. Carson & R.A. Hawkins

*University Department of Surgery, The Royal Infirmary, Edinburgh EH3 9YW, UK.*

**Summary** The oestrogen receptor (ER) is considered to be an essential component of the mechanism of response of a breast tumour to endocrine therapy, but ER measurements have proved to have only modest predictive value. In the present study, we have examined ER status by both immunocytochemical assay (ER-ICA) on a fine needle aspirate and by radioligand-binding assay (DCC) on an excised portion of tumour.

There was a correlation between the ER level detected by the two assays (Spearman's $r = 0.77$ for DCC versus ER-ICA staining intensity, $r = 0.70$ for DCC versus ER-ICA percentage of cells stained, $P < 0.0001$, $n = 137$ in each case). Each assay showed an increasing proportion of ER + ve results with increasing patient age. In the case of ER + ve tissues only, while ER concentration by DCC assay increased steadily with age ($r = 0.39$, $P < 0.0001$, $n = 108$), the ER-ICA assay revealed that, staining intensity increased with age ($r = 0.26$, $P = 0.001$, $n = 149$) but the percentage of cells stained did not ($r = 0.08$, $P$ = NS, $n = 149$).

It is concluded that increasing endocrine responsiveness with advancing age could reflect the increasing proportion of ER + ve tumours with increased levels of ER per cell (as indicated by staining intensity) rather than increasing proportion of ER + ve cells.

Response to endocrine therapy depends, to a large extent, on the presence or absence of oestrogen receptors (Lippman & Allegra 1980; Manni *et al*., 1980; Furr & Jordan, 1984; Hawkins 1985; Oriana *et al*., 1987). In addition, response and oestrogen receptor status have both been reported to change with age (Patterson *et al*., 1981; Beex & Koenders, 1984). Receptor status can be quantified biochemically by the well established radioligand-binding assay or the more recent enzyme-immuno assay (ER-EIA). Using these methods, it has been demonstrated that areas of individual tumours may be heterogeneous for oestrogen receptor expression (Hawkins *et al*., 1980; Crawford *et al*., 1987) as may individual lesions in patients with metastatic disease (Hawkins *et al*., 1981; King *et al*., 1982), so diminishing the ability of biochemical assays to predict the overall response to endocrine therapy. Biochemical assay of tumour cytosols fails to give any information about the cellularity of the lesion or the distribution of the oestrogen receptors at a cellular level. However, by using the immunocytochemical assay (ER-ICA), the localisation and degree of receptor expression can be examined and we have previously demonstrated the importance of heterogeneity in determining whether a patient with a receptor-positive tumour will benefit from treatment with tamoxifen (Gaskell *et al*., 1989). This assay provides two separate pieces of information, namely the intensity of staining and the proportion of cells stained. In the present paper, we have examined the significance of each of these factors in relation to the biochemical assay and to patient age.

### Methods

An immunocytochemical assay for oestrogen receptor (ER-ICA) was carried out on material obtained by fine needle aspiration from 189 patients presenting to the Edinburgh breast clinic with breast cancer. One hundred and thirty-seven of these women underwent surgery, either as the primary treatment (i.e. mastectomy or wide local excision) or underwent wedge biopsy to allow biochemical assay of oestrogen receptors. The mean age of those patients was 67 years (35–86 years). The remainder (52) were treated with tamoxifen without knowledge of their oestrogen receptor status; their mean age was 77 years (70–95).

All patients underwent fine needle aspiration for diagnosis (Dixon *et al*., 1984) and the immunocytochemical assay was carried out on cytospin preparations of the remaining material, as we have described previously (Hawkins *et al*., 1988). In brief, control and test slides were assessed independently by two observers for (a) the percentage of cells stained and (b) the intensity of that staining. The former represents the mean of assessments from multiple fields (preferably > 100 cells per slide) and the latter was assessed on a subjective scale of 0 (none), 1 (weak), 2 (moderate) and 3 (strong), this being aided by reference to the Abbott Quality Control Slide (individual cells mostly staining between 2 and 3), processed with each batch of aspirates. Allowance was made for any non-specific staining in the control slide (usually in the non-malignant cells); a third cytospin preparation was made for each aspirate, air-dried and stained with Giemsa, to provide a check on the cytology.

When each of the two parameters was considered separately for inter-observer agreement, the linear correlation coefficients for the percentage of cells stained and the intensity of staining were 0.957 and 0.930 respectively ($n = 189$, data not shown). Assay precision (coefficients of variation) was calculated using Snedecor's method (Snedecor, 1952) considering the two observers' scores as duplicates; for percentage cells stained and intensity of staining it was 19.6% and 17.2% respectively ($n = 189$, data not shown).

The tumours removed surgically were placed on ice and representative portions were selected by the pathologist for biochemical assay. The assays were carried out using our standard dextran-coated charcoal assay (DCC) for oestrogen receptors, based on competitive binding of a radioligand (tritiated oestradiol) and Scatchard analysis of the results (Hawkins *et al*., 1975, 1981). The oestrogen receptor concentration was calculated as fmol bound $mg^{-1}$ soluble protein. Tumours containing $< 5$ fmol $mg^{-1}$ soluble protein were considered oestrogen receptor-negative and those containing $\geqslant 5$ fmol $mg^{-1}$ soluble protein were considered oestrogen receptor-positive.

### Results

### *Relationship of staining intensity to percentage of cells staining*

These two parameters were very strongly correlated (Spearman's Rank correlation coefficient was 0.85, $P << 0.0001$).

Correspondence: D.J. Gaskell.
Received 3 April 1992; and in revised form 27 May 1992.

*Relationship of ER-ICA staining to ER concentration by DCC assay*

Twenty-seven of 137 tumours were found to be oestrogen receptor-negative when assayed by the DCC assay. Similarly, 40/189 tumours assayed by the ER-ICA assay showed no evidence of immunocytochemical staining for oestrogen receptor protein. When the results obtained by the immunocytochemical assay were compared with the oestrogen receptor concentrations as determined by DCC, there was a good correlation between the results of theDCC assay and the observed intensity of staining (Spearman's Rank correlation coefficient 0.77, $P<0.0001$, $n=137$). When the tumours considered to be oestrogen receptor-positive (by DCC) were considered separately the correlation was maintained, though slightly less strongly (Spearman's Rank correlation coefficient 0.59, $P<0.0001$, $n=108$). The second parameter, the percentage of cells staining, correlated less strongly with the results of the DCC assay (Spearman's Rank correlation coefficient 0.70, $P<0.0001$; for ER + ve tumours only, Spearman's Rank correlation coefficient 0.47, $P<0.001$, $n=108$)). This correlation was not strengthened by combining the two parameters (% cells stained × intensity of staining) to produce a staining index (Spearman's Rank correlation coefficients 0.71, $P<0.0001$, $n=108$ and 0.51, $P<0.0001$, $n=108$ respectively).

*Relationship between patient age and the ER status (DCC and ER-ICA)*

The proportion of oestrogen receptor-negative tumours (as measured by DCC assay) fell steadily with increasing age (Table I). There was a correlation between the median age of the women with oestrogen-positive breast cancers, when they were grouped by decade and the oestrogen receptor concentrations measured in their tumours (Spearman's Rank correlation coefficient 0.39, $P<0.0001$, $n=108$, Figure 1). Similarly, the proportion of tumours failing to show immunocytochemical staining for oestrogen receptor protein fell with increasing age (Table I). Figures 2 and 3 show the relationship between the patient's age and the results of the ER-ICA assay. The intensity of staining correlated moderately well with increasing age (Spearman's Rank correlation coefficient 0.26, $P=0.0001$, $n=149$), but for the oestrogen receptor-positive (staining) tumours the percentage of cells stained did not alter significantly with age (Spearman's Rank correlation coefficients 0.08, $P=$ not significant).

### Discussion

*Inter-relationships between the assays (Table II)*

In the present study, we found that each of the parameters of the ER-ICA assay (percentage cells stained, staining intensity and staining index) showed a good correlation with the results of the biochemical assay. However, three of 29 tumours showing no biochemical evidence of ER-protein showed some immunocytochemical staining and conversely three of the 40 tumours with no evidence of immunocytochemical staining on biochemical assay had significant amounts of ER-protein (⩾ 20 fmol mg$^{-1}$ protein). These findings are

**Table I** Proportion of oestrogen receptor-negative tumours for patients grouped according to age decade

| *Age* | *Receptor negative by DCC assay (< 5 fmol mg$^{-1}$ protein)* | *Receptor negative by ER-ICA assay (no staining)* |
|---|---|---|
| 31–40 | 6/11 (55%) | 7/11 (64%) |
| 41–50 | 8/27 (30%) | 9/27 (38%) |
| 51–60 | 11/45 (24%) | 11/45 (24%) |
| 61–70 | 4/46 (9%) | 4/50 (8%) |
| 71–80 | 0/7 (0%) | 8/42 (19%) |
| 81–90 | 0/1 | 1/13 (8%) |
| 91–100 | | 0/1 |
| Total | 29/137 | 40/189 |

**Figure 1** Relationship between the age of the patient, (grouped by decade) and oestrogen receptor concentration measured in the breast cancer cytosol using the radioligand binding assay. Spearman's Rank correlation coefficient was 0.37, $P<0.0001$, for 108 oestrogen receptor-positive tumours. ⊢—⊣ medians indicated by a horizontal bar.

**Figure 2** Absence of significant relationship between the age of the patient (grouped by decade) and oestrogen receptor status as assessed by the percentage of cells stained immunocytochemically. Spearman's Rank correlation coefficient was 0.08, $P=$ NS, for 149 breast cancers staining for the oestrogen receptor protein. ⊢—⊣ medians indicated by a horizontal bar.

**Figure 3** Relationship between the age of the patient, grouped by decade, and oestrogen receptor status as assessed by the intensity of the immunocytochemical staining. Spearman's Rank correlation coefficient was 0.26, $P<0.001$, for 149 breast cancers staining for the oestrogen receptor protein. ⊢—⊣ medians indicated by a horizontal bar.

**Table II** Summary of inter-relationships between the assays and patient age

| Parameters | | | | Spearman's Rank correlation coefficients for all tumours assayed | Spearman's Rank correlation coefficients for all ER + ve tumours assayed |
|---|---|---|---|---|---|
| DCC | vs | ER-ICA staining intensity | – | 0.77, $P<0.0001$ ($n=137$) | 0.59, $P<0.0001$ ($n=108$) |
| | | ER-ICA % cells stained | – | 0.70, $P<0.0001$ ($n=137$) | 0.47, $P<0.0001$ ($n=108$) |
| | | ER-ICA staining index | – | 0.71, $P<0.0001$ ($n=137$) | 0.51, $P<0.0001$ ($n=108$) |
| % cells stained | vs | ER-ICA staining intensity | – | 0.85, $P<0.0001$ ($n=189$) | 0.61, $P<0.0001$ ($n=149$) |
| Age | vs | DCC | – | 0.47, $P<0.0001$ ($n=137$) | 0.39, $P<0.0001$ ($n=108$) |
| | | intensity of staining | | 0.34, $P<0.0001$ ($n=189$) | 0.26, $P<0.0001$ ($n=149$) |
| | | % cells stained | | 0.24, $P<0.0001$ ($n=189$) | 0.08, $P$ = not significant |

in general agreement with those of other studies comparing biochemical and immunohistochemical/immunocytochemical assays for ER-protein (McCarty *et al.*, 1986; Charpin *et al.*, 1986; Hawkins *et al.*, 1986; Reiner *et al.*, 1986; Weintraub *et al.*, 1987; Horsfall *et al.*, 1989).

*Relationships of the assays to age*

In agreement with earlier workers (Allegra *et al.*, 1979; Beex & Koenders, 1984; Merecki & Jordan, 1985), we have confirmed that breast cancers are more likely to be ER-positive with increasing age and when ER-positive, to contain higher concentrations of ER-protein. The two parameters of the ER-ICA assay (intensity of staining and percentage of cells stained) gave very different results, despite their clear inter-relationship. Although the percentage of cells stained correlated better with response to tamoxifen, as we have previously reported (Gaskell *et al.*, 1989), it was the intensity of staining which had the better correlation with age (Spearman's Rank correlation coefficient 0.26, $P=0.001$, $n=149$).

These findings suggest that the two components of the ER-ICA assay differ in their significance. As the patients get older, the numbers of receptors per cell (intensity of staining) increases, as reflected in the increase in biochemically detected ER concentration with age. Distribution of receptor (% cells stained), however, failed to correlate with age and in our experience and that of other investigators, this is the critical factor in response to endocrine therapy (Pertschuk *et al.*, 1985, Coombes *et al.*, 1987, Gaskell *et al.*, 1989). Our findings may therefore help to explain the difficulties experienced in relating clinical outcome to the absolute oestrogen receptor concentration as measured biochemically in cytosol-based assays (Howat *et al.*, 1983). Tumours containing only a small proportion of cells which stain intensely for ER protein may prove to be relatively resistant to endocrine therapy despite high concentrations of ER protein being detected biochemically; biochemical assays do not take account of tumour cellularity and heterogeneity, as indicated previously by Horsfall *et al.* (1989) using video image analysis of the nuclear optical densities.

**Conclusion**

These data may help to explain why apparently oestrogen receptor-rich breast cancers can fail to respond to endocrine therapy. It is the number of receptors per cell (as indicated by the intensity of staining) which (i) correlates best with the biochemically measured oestrogen receptor level and (ii) changes most with age. However, it is the number (proportion) of receptor-positive cells in a tumour which determines the likelihood of response. This has particular implications for the elderly, where some very high oestrogen receptor concentrations detected biochemically may be of no greater clinical significance than moderate levels detected in younger women.

We thank Professor D.C. Carter for the use of facilities in his Department and his kind advice and support. D.J.G. is grateful to the Breast Cancer Research Trust for support. The radioligand binding assays were performed by A.L.T. and D.C. with funding from the Lothian Health Board. We thank Drs T.J. Anderson, D.A. Patterson and other members of the Department of Pathology for selecting the specimens for analysis and confirming the presence of tumour by histology and cytology.

**References**

ALLEGRA, J.C., LIPPMAN, M.E., THOMPSON, E.B., SIMON, R., BARLOCK, A., GREENE, G., HUFF, K.K., DO, H.M.T. & AITKEN, S.C. (1979). Distribution, frequency and quantitative analysis of oestrogen, progesterone, androgen and glucocorticoid receptors in human breast cancer. *Cancer Res.*, **39**, 1447–1454.

BEEX, L.V.A.M. & KOENDERS, A.J.M. (1984). Is hormonal responsiveness in breast cancer age dependent? *Rev. Endo. Relat. Cancer*, **19**, 5–10.

CHARPIN, C., MARTIN, P.-M., JACQUEMIER, J., LAVAUT, M.N., POURREAU-SCHNEIDER, N. & TOGA, M. (1984). Estrogen receptor immunocytochemical assay (ER-ICA): computerized image analysis system, immunoelectron microscopy, and comparisons with estradiol binding assays in 115 breast carcinomas. *Cancer Res.*, **46** (suppl), 4271–4277.

COOMBES, R.C., POWLES, T.J., BERGER, U., WILSON, P., MCCLELLAND, R.A., GAZET. J.-C., TROTT, P.A. & FORD, H.T. (1987). Prediction of endocrine response in breast cancer by immunocytochemical detection of oestrogen receptors in fine needle aspirates. *Lancet*, **2**, 701–703.

CRAWFORD, D.J., COWAN, S., FITCH, R., SMITH, D.C. & LEAKE, R.E. (1987). Stability of oestrogen receptors in sequential biopsies from patients with breast cancer. *Br. J. Cancer*, **56**, 137–140.

DIXON, J.M., ANDERSON, T.J., LAMB, J., NIXON, S.J. & FORREST, A.P.M. (1984). Fine needle aspiration cytology, in relationship to clinical examination and mammography in the diagnosis of a solid breast mass. *Br. J. Surg.*, **71**, 593–596.

FURR, B.J.A. & JORDAN, V.C. (1984). The pharmacology and clinical use of tamoxifen. *Pharmacol. Therap.*, **25**, 127–205.

GASKELL, D.J., HAWKINS, R.A., SANGSTER, K., CHETTY, U. & FORREST, A.P.M. (1989). Relationship between immunocytochemical estimation of oestrogen receptors in elderly patients with breast cancer and response to tamoxifen. *Lancet*, **1**, 1044–1046.

HAWKINS, R.A., HILL, A. & FREEDMAN, B. (1975). A simple method for the determination of oestrogen receptor concentration in breast tumours and other tissues. *Clin. Chim. Acta*, **64**, 203–210.

HAWKINS, R.A., ROBERTS, M.M. & FORREST, A.P.M.F. (1980). Oestrogen receptors and breast cancer: current status. *Br. J. Srug.*, **67**, 153–169.

HAWKINS, R.A., BLACK, R., STEELE, R.J.C., DIXON, J.M.J. & FORREST, A.P.M. (1981). Oestrogen receptor concentration in primary breast cancer and axillary node metastases. *Breast Cancer Res. Treat.*, **1**, 245–251.

HAWKINS, R.A. (1985). Receptors in the management of breast cancer. *Br. J. Hosp. Med.*, **Sept**, 160–163.

HAWKINS, R.A., SANGSTER, K. & KRAJEWSKI, A. (1986). Histochemical detection of oestrogen receptors in breast carcinoma: a successful technique. *Br. J. Cancer*, **53**, 407–410.

HAWKINS, R.A., SANGSTER, K., TESDALE, A., LEVACK, P.A., ANDERSON, E.D.C., CHETTY, U. & FORREST, A.P.M. (1988). The cytochemical detection of oestrogen receptors in fine needle aspirates of breast cancer; correlation with biochemical assay and prediction of response to endocrine therapy. *Br. J. Cancer*, **58**, 77–80.

HORSFALL, D.J., JARVIS, L.R., GRIMBALDESTON, M.A., TILLEY, W.D. & ORELL, S.R. (1989). Immunocytochemical assay for oestrogen receptor in fine needle aspirpates of breast cancer by video image analysis. *Br. J. Cancer*, **59**, 129–134.

HOWAT, J.M.T., BARNES, D.M., HARRIS, M. & SWINDELL, R. (1983). The association of cytosol oestrogen and progesterone receptors with histological features of breast cancer and early recurrence of disease. *Br. J. Cancer*, **47**, 629–640.

KING, R.J.B., STEWART, J.F., MILLIS, R.R., RUBENS, R.D. & HAYWARD, J.L. (1982). Quantitative comparison of estradiol and progesterone receptor contents of primary and metastatic human breast tumours in relation to their response to endocrine treatment. *Breast Cancer Res. Treat.*, **2**, 339–346.

LIPPMAN, M.E. & ALLEGRA, J. (1980). Quantitative estrogen receptor analysis: the response to endocrine and cytotoxic chemotherapy in human breast cancer and the disease-free interval. *Cancer*, **46**, 2829–2834.

MANNI, A., ARAFAH, B.'U. & PEARSON, O.H. (1980). Estrogen and progesterone receptors in the prediction of response of breast cancer to endocrine therapy. *Cancer*, **46**, 2838–2841.

MCCARTY, K.S., SZABO, E., FLOWERS, J.L., COX, E.B., LEIGHT, G.S., MILLER, L., KONRATH, J., SOPER, J.T., BUDWIT, D.A., CREASMAN, W.T., SEIGLER, H.F. & MCCARTY, K.S. (1986). Use of a monoclonal anti-estrogen receptor antibody in the immunocytochemical evaluation of human tumours. *Cancer Res.*, **46** (suppl), 4244–4248.

MIRECKI, D.M. & JORDAN, V.C. (1985). Steroid receptors and human breast cancer. *Lab. Med.*, **16**, 287–294.

ORIANA, S., SECRETO, G., DI FRONZO, G. & TORRI, A. (1987). Urinary androgens and tumour estrogen receptor as predictors of ovarian response and of survival in advanced breast cancer. *Breast Cancer Res. Treat.*, **9**, 201.

PATTERSON, J.S., BATTERSBY, L.A. & EDWARDS, D.G. (1981). Reviews of the clinical pharmacology and international experience with tamoxifen in advanced breast cancer. *Rev. Endo. Relat. Cancer*, **9** (suppl), 563–582.

PERTSCHUK, L.P., EISENBERG, K.B., CARTER, A.C. & FELDMAN, J.G. (1985). heterogeneity of oestrogen binding sites in breast cancer: morphological demonstration and relationship to endocrine response. *Breast Cancer Res. Treat.*, **5**, 137–147.

REINER, A., SPONA, J., REINER, G., SCHEMPER, M., KOLB, R., KWASNY, W., FUGGER, R., JAKESZ, R. & HOLZNER, J.H. (1986). Estrogen receptor analysis on biopsies and fine needle aspirates from human breast carcinoma. Correlation of biochemical and immunohistochemical methods using monoclonal anti-receptor antibodies. *Am. J. Pathol.*, **125**, 443–449.

SNEDECOR, G.W. (1952). Query No. 92. *Biometrics*, 85–86.

WEINTRAUB, J., WEINTRAUB, D., REDARD, M. & VASSILAKOS, P. (1987). Evaluation of estrogen receptors by immunocytochemistry on fine-needle aspiration biopsy specimen from breast tumours. *Cancer*, **60**, 1163–1172.